# Surgical management of primary mediastinal mature teratoma: A single-center 20 years’ experience

**DOI:** 10.3389/fsurg.2022.902985

**Published:** 2022-09-05

**Authors:** Zhedong Zhang, Xun Wang, Zuli Zhou, Guanchao Jiang, Yun Li

**Affiliations:** Department of Thoracic Surgery, Peking University People's Hospital, Beijing, China

**Keywords:** video-assisted thoracic surgery, surgical treatment, minimally invasive surgical procedures, mediastinum, mature teratoma

## Abstract

**Background:**

This study aims to investigate the clinical efficacy of video-assisted thoracic surgery (VATS) in treating mediastinal mature teratoma (MMT) and explore the clinical factors that increase the difficulties associated with VATS.

**Method:**

We retrospectively reviewed 101 consecutive patients with MMT who underwent surgical excision between November 2001 and June 2021. Follow-up was done by telephone or at an outpatient clinic. The deadline for follow-up was February 2022.

**Results:**

The operative time, the chest tube indwelling time, and the hospital stay duration were significantly shorter in the VATS group compared with the thoracotomy group. The intraoperative and postoperative complication rates in the VATS group were lower than that of the thoracotomy group (*P* < .05). In thoracoscopic surgery, the clinical symptoms during the course of the disease were significantly associated with bleeding loss increasing [odds ratio (OR) = 3.562; 95% confidence interval (CI) 1.180–10.753, *P* = .024] and operation time extension (OR = 5.697; 95% CI 1.529–21.221, *P* = .010). The relationship between lesions and superior vena cava or innominate vein from preoperative CT imaging was significantly associated with bleeding loss increasing (OR = 4.629; 95% CI 1.463–14.639, *P* = .009). A maximal lesion diameter greater than 7 cm was significantly associated with increased risks of operation time extension (OR = 5.019; 95% CI 1.641–15.348, *P* = .005).

**Conclusion:**

Compared with traditional thoracotomy surgery, VATS can be performed safely in selected patients with MMT. A surgical method for complete resection needs to be determined according to preoperative imaging and intraoperative conditions to reduce the unnecessary damage.

## Introduction

Mediastinal teratoma (MT) is one of the most common mediastinal germ cell tumors, with an incidence rate of 5%–10% in primary mediastinal tumors (1). MT often occurs in the anterior mediastinum and contains histologic elements derived from at least two of the three embryonic cell layers: ectoderm, mesoderm, and endoderm. According to histologic differentiation, MT can be divided into mature and immature (2). Mediastinal mature teratoma (MMT) can be cured with complete surgical resection (3). So far, studies on MMT are mostly case reports and few extensive case analyses. Despite the rise of thoracoscopic technology (4), open thoracotomy is still preferred for cases with large tumors. There is still no clear indication in thoracoscopy decisions, including the sizes of the tumor, grades of adhesion, and surgical experience (5). This study aims to clarify the outcomes of video-assisted thoracic surgery (VATS) excision for MMT and to investigate the treatment strategy of MMT with a retrospective analysis over 20 years in our institution. To our knowledge, this is one of the most extensive case series of MMT treated *via* VATS.

## Materials and methods

**Figure 1:**
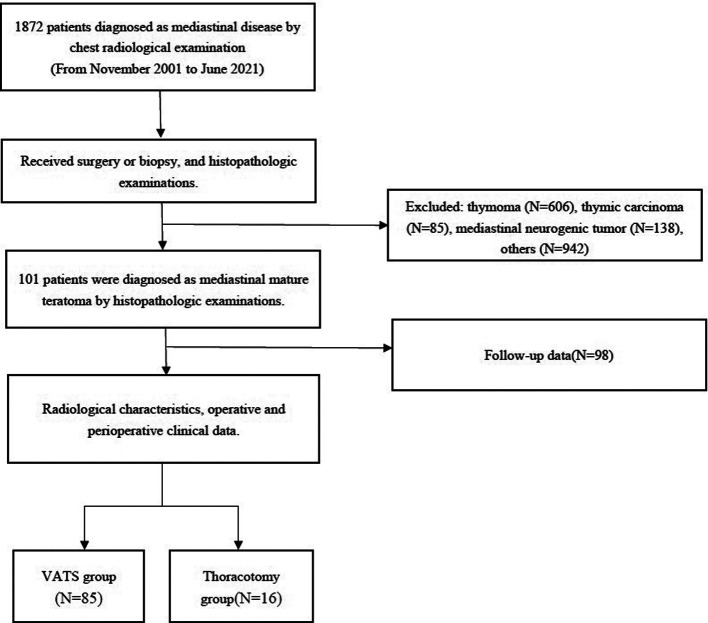
Flow diagram for the patient selection included in this study.

Based on Peking University People's Hospital institutional database, between November 2001 and June 2021, 101 consecutive patients diagnosed as MMT by histopathology after surgical excision were collected. See Figure 1 for a flowchart of the study screening protocol. The criteria for surgical removal of MMT are the following: (1) patients with symptoms caused by the mediastinal mass; (2) gradual enlargement of the mass during clinical observation; (3) preoperative imaging examination cannot exclude malignant tumor; (4) the patient has severe compression of important mediastinal structures, such as the pulmonary artery or main bronchus.

### Preoperative management

**Figure 2:**
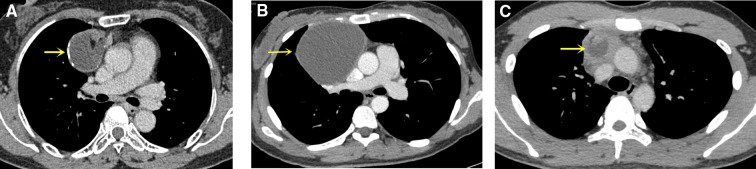
Mediastinal mature teratomas on the CT image. (**A**) Separation is defined as a well-defined lesion, at a certain distance from the surrounding blood vessels (SVC and IV); (**B**) Proximation is defined as a clear boundary between the lesion and surrounding organs, and the blood vessel is partially deformed by the mass; (**C**) Invasion is defined as the non-fatty lesion invaded the surrounding vessels. The yellow arrow points to MMT. CT, computerized tomography; SVC, superior vena cava; IV, innominate vein; MMT, mediastinal mature teratoma.

Chest computerized tomography (CT) scans were used for preoperative diagnosis in all patients. If necessary, magnetic resonance imaging (MRI) or PET-CT could be performed to get additional radiological information. According to the imaging characteristics, we divided the relationship between the MMT and superior vena cava or innominate vein (SVC/IV) found by preoperative CT into three types, including separation, proximation, and invasion (Figure 2): (A) separation is defined as a well-defined lesion, at a certain distance from the surrounding blood vessels; (B) proximation is defined as a clear boundary between the lesion and surrounding organs, and the mass partially deforms the blood vessel; (C) invasion is defined as the non-fatty lesion invaded the surrounding vessels. The clinical symptoms, preoperative imaging data, surgical procedure, and prognosis of these patients were analyzed.

### Operation technique

All patients accepted surgical resection. Surgical resections included thoracoscopic surgery with or without conversion to thoracotomy, semi-clamshell, median thoracotomy, simple neck collar incision, and posterolateral thoracotomy, which were performed according to the relationship between the surrounding tissue and tumor location, tumor size, invasion, and pleural adhesions.

After preoperative preparation, patients were under general anesthesia with one-lung ventilation by double-lumen endotracheal intubation. VATS technique was initially performed for selected patients. The surgical procedures were conducted in the 30°–45° lateral recumbent position with a three-port or two-port approach. The camera port (1 cm) was first created in the fifth intercostal space on the mid-axillary line. Under the direct thoracoscopic vision, the operator port (4 cm) and the assisted-operator port (1 cm) were selected according to the site of the teratoma. The operator port usually was made on the midclavicular line at the fourth to fifth intercostal spaces and the assisted-operator port was made on the anterior axillary line at the third intercostal spaces. The assisted-operator port might be omitted for simple cases.

When the tumor was densely attached to the surrounding tissues and could not be resected under the endoscope, or damaged the IV when mobilizing the MMT, conversion to thoracotomy was performed. The operation port was extended to 15 cm and connected to the camera hole, and the fourth sternocostal joint was cut to form a semi-clamshell incision at the lower margin of the breast. If the mass is located in the middle of the anterior mediastinum, adequate exposure of the hilar and thoracic structures on the surgical side is not required, direct median thoracotomy was chosen for patients whose preoperative operation evaluation is too difficult to perform under the endoscope. A simple cervical incision is used if the MMT is located higher, below the thyroid. Difficulty operation was defined as the operation time exceeding 120 min or the intraoperative blood loss more significant than 200 ml.

Our surgical principles are the following: (1) the teratoma should be resected entirely as far as possible, and the extravasation of cystic contents should be avoided; (2) when complete excision could not be performed for severe adhesions, the residual part of the tumor should be destroyed by chemical agents or physical damage; (3) when thymoma or other malignant tumors of the thymus could not be excluded according to preoperative examination or intraoperative exploration, thymectomy should be performed (6). At the end of the surgery, one 24 Fr drainage tube was placed in the thoracic cavity.

Histologic examinations confirmed the diagnosis of mature teratoma based on the existence of multilayer epithelial cells and their appendages such as hair follicles, sebaceous glands, sweat glands on the cystic wall, and three layers of various tissue components microscopically.

### Postoperative follow-up

All patients accepted scheduled follow-up done by telephone or at an outpatient clinic. The postoperative complications were documented, and the deadline for follow-up was February 2022.

### Statistical analysis

Statistical analyses were performed using the SPSS version 23.0 package (IBM Corporation, Armonk, NY, USA). Descriptive analyses were presented as median ± standard deviation for normally distributed variables and median or ranges for non-normally distributed variables. The Student's *t*-test was used for the comparisons of normally distributed variables, and the Mann–Whitney *U* test was used for comparing non-normally distributed variables. Categorical variables were compared by the *Chi-square* test.

Univariate and multivariable logistic regression analyses were used to identify independent predictors for operative difficulty. Predictors (*P* < 0.1) in the univariate analysis would be included in a multivariable analysis. *P*-values <.05 were considered statistically significant.

The study was approved by the ethics committee of the Department of Thoracic Surgery at Peking University People's Hospital. Written consent was obtained from all patients before surgery.

## Results

### Clinical characteristics

A total of 63 female and 38 male patients had an average age of 33.1 ± 14.2 years (range 4–72 years). Among these patients with mature teratomas, 58 cases (57.4%) were asymptomatic at the first time of diagnosis by radiological examinations incidentally. The most common symptoms included chest pain (27.7%), chest tightness or discomfort (10.9%), and other symptoms, including cough (3.0%) and fever (1.0%). There were 100 teratomas located in the anterior mediastinum and one in the posterior mediastinum. All the patients' preoperative laboratory tests for alpha-fetoprotein (AFP) and beta-human chorionic gonadotropin (β-hCG) levels were at normal levels. According to the methods of the operation, we divided the patients into two groups: the VATS group (the whole course of the procedure was completed by thoracoscopy) and the Thoracotomy group (the process was completed by thoracotomy, including VATS conversion to thoracotomy). Only 77.2% (78/101) of patients were correctly diagnosed with teratoma by CT before surgery, and the rate was 81.3% (13/16) in the thoracotomy group and 76.5% (65/85) in the VATS group. Other radiographic diagnoses included mediastinal cyst in six patients, thymoma in six patients, mediastinal abscess in two patients, pericardium cyst in one patient, and mediastinal mass with undefined diagnosis in eight patients. No specific pathological diagnoses were made before surgery. The demographic and preoperative data of these patients are shown in Table 1. And there were significant differences between the VATS group and the thoracotomy group in gender, clinical symptoms, tumor diameter, and details of the mass and vessels shown on preoperative CT (*P* < .05).

**Table 1:** Demographic and preoperative data of patients with mediastinal mature teratoma.

Variables	ALL	VATS	Thoracotomy^a^	*t*/*χ*^2^*/U*	*P*-value
	(*N* = 101)	(*N* = 85)	(*N* = 16)		
Age (mean ± SD, range)	33.1 ± 14.2 (4–72)	32.9 ± 13.7	33.8 ± 17.0	0.224	0.823
Sex (male/female)	38/63	27/58	11/5	7.849	.005
Body mass index (mean ± SD, range)	23.0 ± 4.0	23.0 ± 3.87	23.1 ± 4.7	0.059	0.953
Clinical symptom (%)	43 (42.6%)	31 (36.5%)	12 (75.0%)	13.484	.004
No symptom	58 (57.4%)	54 (63.5%)	4 (25.0%)		
Chest pain	28 (27.7%)	19 (22.4%)	9 (56.3%)		
Chest tightness or discomfort	11 (10.9%)	10 (11.8%)	1 (6.3%)		
Fever	1 (1.0%)	0	1 (6.3%)		
Cough	3 (3.0%)	2 (2.4%)	1 (6.3%)		
Clinical symptom duration (days, median, range)	60 (2–3,650)	60 (2–3,650)	75 (7–730)	626.000	0.614
Tumor maximum size (cm, mean ± SD, range)	7.8 ± 3.4 (1–19)	7.3 ± 3.0 (1–14.3)	10.7 ± 3.8 (5–19)	3.866	.000
Diagnosed as mediastinal mature teratoma by CT (%)	78 (77.2%)	65 (76.5%)	13 (81.3%)	0.009	0.926
Relationship between lesions and SVC or IV from preoperative CT imaging (%)				32.689	.000
Separation	48 (47.5%)	47 (55.3%)	1 (6.3%)		
Proximation	26 (25.7%)	25 (29.4%)	1 (6.3%)		
Invasion	27 (26.7%)	13 (15.3%)	14 (87.5%)		
Tumor types (%)				1.743	0.418
Cystic	25 (24.8%)	23 (27.1%)	2 (12.5%)		
Solid-cystic	70 (69.3%)	57 (67.1%)	13 (81.3%)		
Solid	6 (5.9%)	5 (5.9%)	1 (6.3%)		

SD, standard deviation; CT, computerized tomography; SVC, superior vena cava; IV, innominate vein; VATS, video-assisted thoracic surgery.

^a^ Included conversion to thoracotomy.

### Surgical results

The surgical methods of the 101 patients are summarized in Table 2. Furthermore, six patients were VATS converted to thoracotomy. Among them, two patients were actively converted to thoracotomy because of difficulty in surgery; the remaining four were passively due to intraoperative injury of the IV. The essential data, preoperative imaging data, and intraoperative details of these six patients are summarized in Table 3.

**Table 2:** Type of surgical methods in patients with mediastinal mature teratoma.

**Surgical methods**			
VATS	85	**VATS conversion to thoracotomy**	6
Median thoracotomy	1	Hemi-clamshell approach	6
Hemi-clamshell approach	12		
Posterolateral incision	2		
Simple neck collar incision	1		
**Total**	101		

VATS, video-assisted thoracic surgery.

**Table 3:** Details of patients converted to thoracotomy.

Patient	Age (years)	Sex	BMI	Clinical symptom	Tumor maximum size (cm)	Preoperative CT imaging	Intraoperative exploration	Cause of conversion to thoracotomy
Patient 1	53	Male	32.11	Chest tightness	5	The non-fatty lesion invaded the IV	The mass invaded the IV and was closely related to the surrounding tissues	Surgery could not be completed under VATS
Patient 2	34	Male	21.01	NA	8	The non-fatty lesion invaded the SVC and pericardium	A large area of adhesion between the tumor and the pericardium, with a high upper pole	The mass densely adhered to the pericardium, and the pericardium is repaired by thoracotomy
Patient 3	20	Female	19.03	Cough	14.3	The non-fatty lesion invaded the IV and peripheral lung tissue	The mass was closely related to the IV and adjacent lung tissue	IV injured during the dissociation of mass, conversion to thoracotomy for blood vessels repairing
Patient 4	31	Male	24.91	Chest tightness	10.5	The non-fatty lesion invaded the IV and peripheral lung tissue	The mass was closely related to the IV and left upper lobe lung	IV injured during the dissociation of mass, conversion to thoracotomy for blood vessels repairing
Patient 5	72	Female	20.66	NA	8	The non-fatty lesion invaded the IV	The mass was closely related to the IV	IV injured during the dissociation of mass, conversion to thoracotomy for blood vessels repairing
Patient 6	30	Male	26.12	Chest tightness	14	The non-fatty lesion invaded the IV	The mass was closely related to the IV and phrenic nerve	IV injured during the dissociation of mass, conversion to thoracotomy for blood vessels repairing

BMI, body mass index; VATS, video-assisted thoracic surgery; IV, innominate vein; BMI, body mass index; SVC, superior vena cava.

The average operation time was 171.0 ± 9.1 min (range, 60–600 min), and the median intraoperative blood loss was 60 ml (range, 10–2,600 ml). The median postoperative drainage tube removal time was 2 days (range, 1–31 days). The postoperative hospital stay was 5 days (range, 2–41 days). In seven patients with MMT, the resection was incomplete because the teratoma severely adhered to the SCV/IV, pericardium, and lung tissue, and the tumor could not be completely freed. The pericardial injury occurred in one patient, phrenic nerve injury in four patients, and IV injury in four patients during surgery. There were no perioperative period deaths (within 30 days of surgery). After symptomatic treatment, the patients improved. VATS was associated with less intraoperative blood loss and intraoperative complications; postoperative complications, drainage tube removal time, and length of hospital stay were shorter than thoracotomy (*P* < .05) (Table 4). After the operation, 101 cases were followed up, with three cases being lost to follow-up. The follow-up time was 12–243 months. None of the patients had relapses during follow-up.

**Table 4:** Perioperative and postoperative results of the patients with mediastinal mature teratoma.

Clinical Data	ALL	VATS	Thoracotomy	*t*/*χ*^2^/*Z*	*P*-value
	(*N* = 101)	(*N* = 85)	(*N* = 16)		
Operation time (min, mean ± SD, range)	171.0 ± 9.1 (60–600)	151.3 ± 65.8 (60–380)	275.9 ± 133.3 (65–600)	3.657	.002
Intraoperative blood loss (ml, median, range)	60 (10–2,600)	50 (10–700)	750 (10–2,600)	−5.364	.000
Surgery-related complications (%)	9 (8.9%)	2 (2.4%)	7 (43.8%)	23.558	.000
Pericardial injury	1 (6.3%)	0	1 (6.3%)		
Phrenic nerves injury	4 (4.0%)	2 (2.4%)	2 (12.5%)		
Innominate vein injury	4 (4.0%)	0	4 (25.0%)		
Incomplete resection of the teratoma (%)	7 (6.9%)	4 (4.7%)	3 (13.8%)	2.228	0.136
The extent of surgery resection (%)				1.768	0.622
MMT	7 (6.9%)	5 (5.9%)	2 (12.5%)		
MMT+PT	48 (47.5%)	40 (47.1%)	8 (22.2%)		
MMT+T	37 (36.6%)	33 (38.8%)	4 (25.0%)		
MMT+T+PLT	9 (8.9%)	7 (8.2%)	2 (12.5%)		
Chest tube duration (days, median, range)	2 (1–31)	2 (1–7)	4 (2–31)	−3.962	.000
Postoperative hospital stays (days, median, range)	5 (2–41)	4 (2–11)	8 (4–41)	−3.487	.000
Postoperative complications (%)	7 (6.9%)	3 (3.5%)	4 (25.0%)	9.751	.021
Pneumonia	1 (1.0%)	0	1 (6.3%)		
Hydrothorax	5 (5.0%)	3 (3.5%)	2 (12.5%)		
Chylothorax	1 (1.0%)	0	1 (6.3%)		

PT, partial thymus; T, thymus; PLT, partial lung tissue; MMT, mediastinal mature teratoma; SD, standard deviation; VATS, video-assisted thoracic surgery.

Univariate and multivariate logistic regression analyses were performed to identify factors that may increase the operative time (more than 120 min) and intraoperative bleeding (more than 200 ml) in VATS surgery. Multivariate analysis showed that the largest diameter greater than 7 cm [odds ratio (OR) = 5.019; 95% confidence interval (CI) 1.641–15.348, *P* = .005] and clinical symptoms during the course of the disease (OR = 5.697; 95% CI 1.529–21.221, *P* = .010) were significantly associated with longer operative time (Table 5). In addition, multivariate analysis showed that clinical symptoms (OR = 3.562; 95% CI 1.180–10.753, *P* = .024) and preoperative chest CT showed a strong relationship between teratoma and SVC/IV (OR = 4.629; 95% CI 1.463–14.639, *P* = .009) were significantly associated with an increased risk of blood loss (Table 6).

**Table 5:** Univariate and multivariate analysis of risk factors for increasing operation time.^a^

Variables	Univariate analysis			Multivariate analysis		
	OR	95% CI	*P*-value	OR	95% CI	*P*-value
Age (<40 vs. ≥40 years)	0.875	0.317–2.413	0.875			
Sex (female vs. male)	1.480	0.504–3.608	0.552			
BMI (<23 vs. ≥23)	1.056	0.412–2.704	0.910			
Clinical symptom (yes vs. no)	5.819	1.791–18.908	0.003	5.697	1.529–21.221	.01
Preoperative follow-up time (≤2 vs. >2 years)	0.354	0.074–1.702	0.195			
Tumor types (non-solid vs. solid/part-solid)	0.312	0.095–1.026	0.055	0.362	0.087–1.506	0.163
Diagnosed as mediastinal mature teratoma by CT (yes vs. no)	0.783	0.265–2.312	0.657			
Relationship between lesions and SVC or IV from preoperative CT imaging (separation vs. proximation/invasion)	3.029	1.149–7.982	0.025	2.639	0.807–8.624	0.108
Surgical approach (right vs. left)	1.007	0.409–2.482	0.987			
Extent of surgery resection (MMT ± PT vs. MMT + T + PLT)	0.778	0.316–1.915	0.585			
Complete resection of mass (yes vs. no)	0.000	0.000	0.999			
Maximal diameter (≤7 vs. >7 cm)	6.074	2.270–16.253	0.000	5.019	1.641–15.348	.005

PT, partial thymus; T, thymus; PLT, partial lung tissue; MMT, mediastinal mature teratoma; SVC, superior vena cava; IV, innominate vein; CT, computerized tomography; OR, odds ratio; CI, confidence interval; BMI, body mass index.

^a^ The operation time prolonged is defined as the operation time was more than 120 min.

**Table 6:** Univariate and multivariate analysis of risk factors for increased blood loss.^a^

Variables	Univariate analysis			Multivariate analysis		
	OR	95% CI	*P*-value	OR	95% CI	*P*-value
Age (<40 vs. ≥40 years)	0.796	0.254–2.493	0.695			
Sex (female vs. male)	0.548	0.178–1.686	0.294			
BMI (<23 vs. ≥23)	0.609	0.210–1.722	0.363			
Clinical symptom (yes vs. no)	3.611	1.315–9.920	0.013	3.562	1.180–10.753	.024
Preoperative follow-up time (≤2 vs. >2 years)	0.452	0.051–3.983	0.475			
Tumor types (non-solid vs. solid/part-solid)	1.943	0.580–6.514	0.282			
Diagnosed as mediastinal mature teratoma by CT (yes vs. no)	0.762	0.251–2.313	0.631			
Relationship between lesions and SVC or IV from preoperative CT imaging (separation vs. proximation/invasion)	4.97	1.702–14.514	0.003	4.629	1.463–14.639	.009
Surgical approach (right vs. left)	1.232	0.461–3.295	0.678			
Extent of surgery resection (MMT ± PT vs. MMT + T + PLT)	1.29	0.480–3.469	0.614			
Complete resection of mass (yes vs. no)	0.102	0.010–1.040	0.054	0.308	0.026–3.639	0.308
Maximal diameter (≤7 vs. >7 cm)	1.714	0.615–4.781	0.303			

OR, odds ratio; CI, confidence interval; BMI, body mass index; CT, computerized tomography; PT, partial thymus; T, thymus; PLT, partial lung tissue; SVC, superior vena cava; IV, innominate vein; MMT, mediastinal mature teratoma.

^a^ The blood loss increased is defined as the blood loss is more than 200 ml.

## Discussion

MMT, due to its own characteristics, may have no obvious clinical symptoms for its small size in the early stage, so it is more often found by chance during physical examination. With the increase of age, the enlarged tumor is often found by examination due to compression of surrounding organs, secondary infection, or corresponding clinical symptoms when penetrating the surrounding tissue. At present, the diagnosis mainly relies on chest CT to see calcification in the tumor, and even teeth or bones (7). However, most MMT is cystic or cystic-solid masses, easily misdiagnosed as thymoma before surgery. Only 77.2% of the patients with a mediastinal mass can be correctly diagnosed as MMT before surgery in our center. Surgical resection is the only effective treatment for MMT (8).

With the development of thoracoscopy technology for more than 20 years and the advancement of medical devices, VATS has obvious advantages such as minor trauma, less intraoperative blood loss, shorter postoperative hospital stays, and faster recovery than thoracotomy. Therefore, the indications of thoracoscopic surgery have gradually expanded, and it basically covers all fields of modern thoracic surgery (9). For mediastinal tumors, which mainly occur in young people, this group of people has higher aesthetic requirements, so thoracoscopy is more widely used in mediastinal tumor resection. Until now, the largest series of endoscopic treatment of MT is only 22 cases (10); the first case of thoracoscopic MMT resection in our center was carried out in 2001, and 87 cases of thoracoscopic MMT resection were completed in the past 20 years. At present, thoracoscopy mainly adopts the right-side approach. For tumors that are biased to the left, the left thoracic incision or the subxiphoid system can be used. Compared with its advantages, its disadvantage is the poor exposure of the lateral mediastinum and superior mediastinum, especially when the tumor is large or has heavy adhesion to the surrounding tissue, which often leads to incomplete tumor resection, and it is easy to accidentally injure bilateral phrenic nerves and internal mammary artery and IV (11). Among the thoracotomy criteria for anterior mediastinal tumor resection, such as severe compression of important mediastinal structures, median sternotomy has a clear surgical field, which can calmly face various unexpected situations that may occur during surgery and even add extracorporeal circulation and other advantages. In our center, one case of MMT was treated with median sternotomy. However, sternotomy also has certain limitations. The structure of the thoracic cavity and hilum of the operation side is poorly exposed, the damage is extensive, the operation is complicated, the equipment requirements are high, and the probability of sternal complications is also high. For complicated MMT, a Hemi-clamshell incision is mainly used in our center. Its advantage is that the mediastinum and the important viscera of the hilar lung are under the direct view of the surgeon during the operation. When necessary, the sternum can be transected to a clamshell incision to take various emergency measures such as extracorporeal circulation. Low surgical instrument requirements and no need for swinging saws can also avoid sternum infection and sternum nonunion complications. At the same time, if the tumor did invade the SVC and IV, and the tumor could not be completely removed during the operation, we would choose to send the intraoperative frozen pathology. If the pathology indicated a benign condition, incomplete resection would be taken after full communication with the patient's family to ensure the safety of the operation, and a regular postoperation review would be conducted. If the pathology indicates malignancy and vascular replacement is needed, the sternum can be transected to make a clamshell incision. At the same time, if thoracoscopic exploration is required first, the patient only needs a 30–45° lateral decubitus position. When a thoracotomy is required, the patient can directly switch to a recumbent and semi-clamshell incision. Therefore, median sternotomy is rarely used for MMT in our center.

The surgical indication for thoracoscopy is usually defined as a mediastinal mass less than 6 cm (12). Although some centers have expanded the indication for thoracoscopic tumor resection, it is still smaller than 10 cm (1). At the same time, other factors besides tumor size, such as intraoperative exploration of the tumor, the surgeon's experience with thoracoscopy, available equipment and support devices, can also affect the decision of thoracoscopic surgery indications. Our findings suggest that the presence or absence of clinical symptoms and preoperative analysis of computed tomography (tumor size and tumor boundary with essential blood vessels) are two critical indicators of surgical difficulty for MMT. The difficulty in resection of giant MT is that the tumor occupies the thorax and the surgical field is poorly exposed, so it is easy to damage the surrounding important tissues. In our center, the CT size of the largest tumor removed by thoracoscopic surgery was 14.0 cm × 6.0 cm × 6.0 cm, with an average diameter of 7.3 cm. Attention should be paid during the operation: (1) thoracoscopic exploration can be performed first, and the tumor volume can be reduced by endoscopic operation; for cystic lesions, the cyst wall can be punctured and the cyst fluid can be sucked out to provide a wider thoracoscopic field of view, making it easier to remove completely (11); for larger solid tumors, part of the contents can be resected in the capsule to shrink the tumor and obtain more adequate processed after exposure; (2) if the operation under VATS is difficult and the tumor and surrounding adhesions are dense, the surgical incision should be enlarged as much as possible.

MMTs are distinguished from other benign mediastinal tumors by their aggressive growth characteristics. For symptomatic patients diagnosed with MMT, the boundary between the tumor and surrounding tissues is often unclear, and severe adhesions are prone to occur, so the surgical difficulty is also increased. At the same time, studies have shown no difference between the VATS group and the thoracotomy group in patients with teratoma in contact with the left brachiocephalic vein on preoperative CT, indicating the adhesion of the tumor and surrounding organs may be more critical than the tumor location (5). Chang et al. found that thoracoscopic surgery is feasible if no dense adhesions are found on preoperative radiographic evaluation, and symptoms may be a relative contraindication to thoracoscopic surgery because of its association with surgical complications (13). The deficiency of the study is that degree of dense adhesion is not clearly defined. Our study classified the degree of adhesion of the teratoma to the vital blood vessels, namely the SVC and the brachiocephalic vein, into three categories, namely, distant, compressive, and invasion. Multivariate analysis found that when the relationship between the tumor and vital blood vessels was compression and invasion, the difficulty of operation (increased intraoperative blood loss) increased significantly. In our center, six patients had converted to thoracotomy, of which two patients had converted to thoracotomy actively, and four patients had converted to thoracotomy passively due to intraoperative injury of the IV. The main reasons for the conversion of thoracotomy during thoracoscopic thoracotomy reported in other centers are (1) tight adhesion to surrounding tissues (pericardium, lung, phrenic nerve, and vital blood vessels), and the operation cannot be completed by VATS (14); (2) intraoperative damage to essential structures (pericardium, vital blood vessels, etc.) need to be opened for repair (15). Therefore, if combined with the patient's clinical symptoms before surgery and the imaging data suggest that the tumor is large and densely adhered to the surrounding area, it can be converted to minor incision surgery after endoscopic exploration to ensure the safety of the surgery.

Most teratomas are located in the anterior mediastinum near the bottom of the pericardium and connect with the residual tissue of the thymus (16). Teratomas may contain the pancreas, salivary glands, and other tissues to secrete digestive enzymes that act on surrounding tissues and are easy to form adhesions or perforations with adjacent tissues and organs. Its external penetration range includes the pleural cavity, pericardial cavity, lung, neck, and great vessels (17). Therefore, for an MMT with a smaller diameter, intact capsule, and no serious tissue adhesion, only the tumor and part or all of the thymus need to be removed entirely. When the tumor is huge, preoperative CT combined with intraoperative exploration reveals an invasion of adjacent tissues. (1) Lung: A double-lumen tube is inserted into the trachea during the operation to prevent contralateral aspiration or sowing during the operation (18). Whether or not to perform lobectomy at the same time should be determined according to the specific circumstances of the tumor invading the lung. Pulmonary resection was performed in nine cases in our center. Two cases were decided to undergo right upper lobectomy due to severe adhesion to the right upper lobe and loss of function of the right upper lobe. The remaining seven cases had local adhesion invasion, and wedge resection was the main method. (2) Pericardium: Partial resection of the pericardium can be performed when the tumor invades the pericardium. In our center, there was one case of MMT and teratoma with severe adhesion. After the tumor and partial pericardium resection were performed under VATS, the pericardium was repaired by converting to thoracotomy. (3) IV/SVC: When the tumor invades the IV and SVC, more elaborate actions are required during the operation to avoid rupture and bleeding of blood vessels. If the tumor and blood vessels are closely adhered to or infiltrated, angioplasty or artificial blood vessel replacement may be considered.

There are few reports on the coexistence of malignant components in mature teratomas (19). In seven patients in our center, the tumor could not be completely removed due to the severe adhesion of the tumor to the SVC, IV, lung tissue, and pericardium. No recurrence or metastasis was found in postoperative follow-up. It has been reported in previous literature that when the residual tumor wall of MMT is infiltrated with large blood vessels and is difficult to separate, if the intraoperative pathology confirms that the tumor is benign, in order to reduce the incidence of intraoperative secondary injury. After scraping the tumor wall endothelium, iodine or carbolic acid should be applied. Regular follow-up after surgery is recommended (8).

Our study has several limitations. First, although the study spans a long time, it is still a single-center retrospective study, and selection bias is inevitable, so there may be a lack of homogeneity among different groups of patients. To confirm the findings, it is worth conducting multicenter studies in more MMT patients. Second, with the increase in the volume of single-center mediastinal mass operations, the surgical techniques may become more proficient, and the indications for surgical thoracoscopic surgery for teratoma will expand accordingly.

This is the single-center study with the largest sample size of VATS in mature teratomas to date. In our 20-year experience with mediastinal mature teratomas, we determined that VATS resection is a feasible choice, with a shorter postoperative recovery time if there are no dense adhesions present in the preoperative CT scan. For large tumors and preoperative clinical symptoms, when the relationship between the tumor and surrounding blood vessels is found to be oppressed or invaded by preoperative imaging combined with intraoperative exploration, in order to ensure the safety of the operation, conversion to thoracotomy after VATS exploration can be considered. For patients with incomplete resection, although the intraoperative pathology proved to be benign, regular follow-up is required after surgery.

## Data Availability

The raw data supporting the conclusions of this article will be made available by the authors, without undue reservation.
